# Hindering and facilitating factors in the implementation of digital mental health interventions within community settings

**DOI:** 10.1192/j.eurpsy.2024.1148

**Published:** 2024-08-27

**Authors:** K. Turmaine, K. Chevreul

**Affiliations:** ^1^ECEVE1123, Inserm/Université de Paris Cité; ^2^ECEVE1123, URC Robert Debré, Paris, France

## Abstract

**Introduction:**

The digitalisation of the society has made inevitable the development and use of digital health. In mental health care, the use of digital tools has been questioned, although their capacity to improve accessibility to evidence-based information and tackle stigma has been recognised. The paradox of these virtual tools is that they need to rely on local resources to get used and disseminated.

**Objectives:**

To identify the factors from the context that could help or hinder the set-up of an effective intervention in digital mental health.

**Methods:**

Between 2018 and 2020, a digital mental health intervention, based on the promotion of StopBlues, a digital tool targeting psychic distress and suicide in in the adult general population, was conducted in 32 willing French localities. In each of the latter, a focal person was designated among the officials to organise the promotion locally and liaise with the research team. Employing interviews and observations, we identified the factors from the context that were favouring or hindering the intervention.

**Results:**

The qualitative approach unveiled the existing dynamics between local stakeholders and difficulties faced by the focal persons. It appeared that the pollical context particularly influenced the outcome of the intervention. In parallel, the endorsement by local hospitals and psychiatrists was equally crucial confirming the key role they play when they champion a cause at the forefront.

**Conclusions:**

Real-world evaluations using both qualitative and quantitative methods of digital mental health interventions have to be implemented in order to understand how they can help people. If these interventions are in line with the 1986 Ottawa Charter in terms of patient empowerment, they still need to be supported by local stakeholders, both at the pollical and medical levels.

**Disclosure of Interest:**

None Declared

